# Clinical manifestations and imaging and pathological features of giant cell angioblastoma: Report of four cases and literature review

**DOI:** 10.3389/fsurg.2022.1062309

**Published:** 2023-01-05

**Authors:** Qingbin Li, Zhaohua Zhang, Guocai Chen, Yongbo Hu, Rongjun Mao, Le Xie, Shaoluan Chen, Yongqiang Lao, Junqing Gao

**Affiliations:** ^1^ Department of Orthopaedics, The Eighth Clinical Medical College of Guangzhou University of Chinese Medicine, Foshan, China; ^2^ Department of Orthopaedics, Foshan Hospital of Traditional Chinese Medicine, Foshan, China; ^3^ Department of Pathology, Foshan Hospital of Traditional Chinese Medicine, Foshan, China; ^4^ Department of Radiology, Foshan Hospital of Traditional Chinese Medicine, Foshan, China

**Keywords:** vascular neoplasm, giant cell angioblastoma, bone tumour, immunohistochemistry, GCAB

## Abstract

Giant cell angioblastoma is a relatively rare vasogenic tumour. To date, studies on its clinical manifestations, imaging characteristics, pathological features, and prognosis are extremely limited and unknown, with only a few cases recorded. In this study, four cases of giant cell angioblastoma confirmed by pathological examination were reported to improve our understanding and deep exploration of the tumour spectrum. All cases in our study were male, including two adults and two boys. The lesions were located in the lower segment of the femur, medial condyle of the femur, knee joint, and popliteal fossa. Regarding the imaging characteristics, two patients with lesions in bone showed bone destruction, while the other two had lesions that invaded soft tissues, showing irregular, abnormal signal shadows and obvious enhancement. Histopathological analysis revealed that the nodular tumour tissue was mainly composed of oval and spindle cells, with varying numbers of osteoclast-like multinucleated giant cells, and the interstitial tissues were often filled with blood vessels of different sizes. The immunophenotype demonstrates that endothelial cells of small vessels in nodules expressed CD31, SMA, and ERG, while osteoclast-like multinucleated giant cells and histiocytes expressed CD68 and CD163, and the surrounding cells expressed SMA. All four patients were treated with surgical resection. One of them relapsed 1 month after surgery and received a second surgical resection. No distant metastasis or death occurred during the follow-up period. This study indicates that giant cell angioblastoma is a local invasive vascular tumour that can develop both in children and adults with skin, mucous membrane, soft tissue, and bone involvement. Imaging characteristics show bone destruction and irregular, abnormal signal shadows; in addition, obvious pathological morphological features can be observed. Currently, the treatment is mainly surgical resection, and interferons may be used as adjuvant chemotherapy.

## Introduction

Giant cell angioblastoma (GCAB) is a rare vascular tumour that was first reported in 1991 by Gonzalez-Crussi et al. (1). To our knowledge, only 12 cases have been reported, including 7 men and 5 women ranging from adults to infants, and the oldest case is 56 years old. GCAB is an invasive tumour with skin, mucous, bone, and soft tissue involvement (1–6); however, the clinical manifestations, imaging characteristics, pathological features, and prognosis of the disease are still unclear. As a result, it has not yet been regarded as a new tumour classification in the latest WHO Classification of Tumours of Soft Tissue and Bone and has been temporarily included in intermediate local invasive vascular tumours (7). In this study, four cases diagnosed by pathology were reported, and the imaging manifestations and pathological features of GCAB were comprehensively analysed in combination with the published literature, aiming to improve the understanding and accuracy of diagnosis.

## Materials and methods

Data on four cases of giant cell angioblastoma diagnosed in Foshan Hospital of Traditional Chinese Medicine from 2011 to 2021 were collected. The follow-up time ranged from 0.5 to 10 years. The clinical data, imaging data, and pathological results of all patients were provided by the inpatient data system.

After surgical resection, all samples were fixed with a 10% neutral formaldehyde solution and dehydrated with ethanol immediately. Then, the paraffin-embedded pathology samples were sectioned to 4 μm, stained with haematoxylin and eosin, and observed with low- and high-magnification microscopes. Meanwhile, continuous sections were marked by immunohistochemistry, and the envisioned two-step method was adopted. The selected antibodies included CD31, F8, CD34, ERG, FLi-1, SMA, calponin, desmin, CD68, and CD163.

## Results

### Case 1

**Figure 1:**
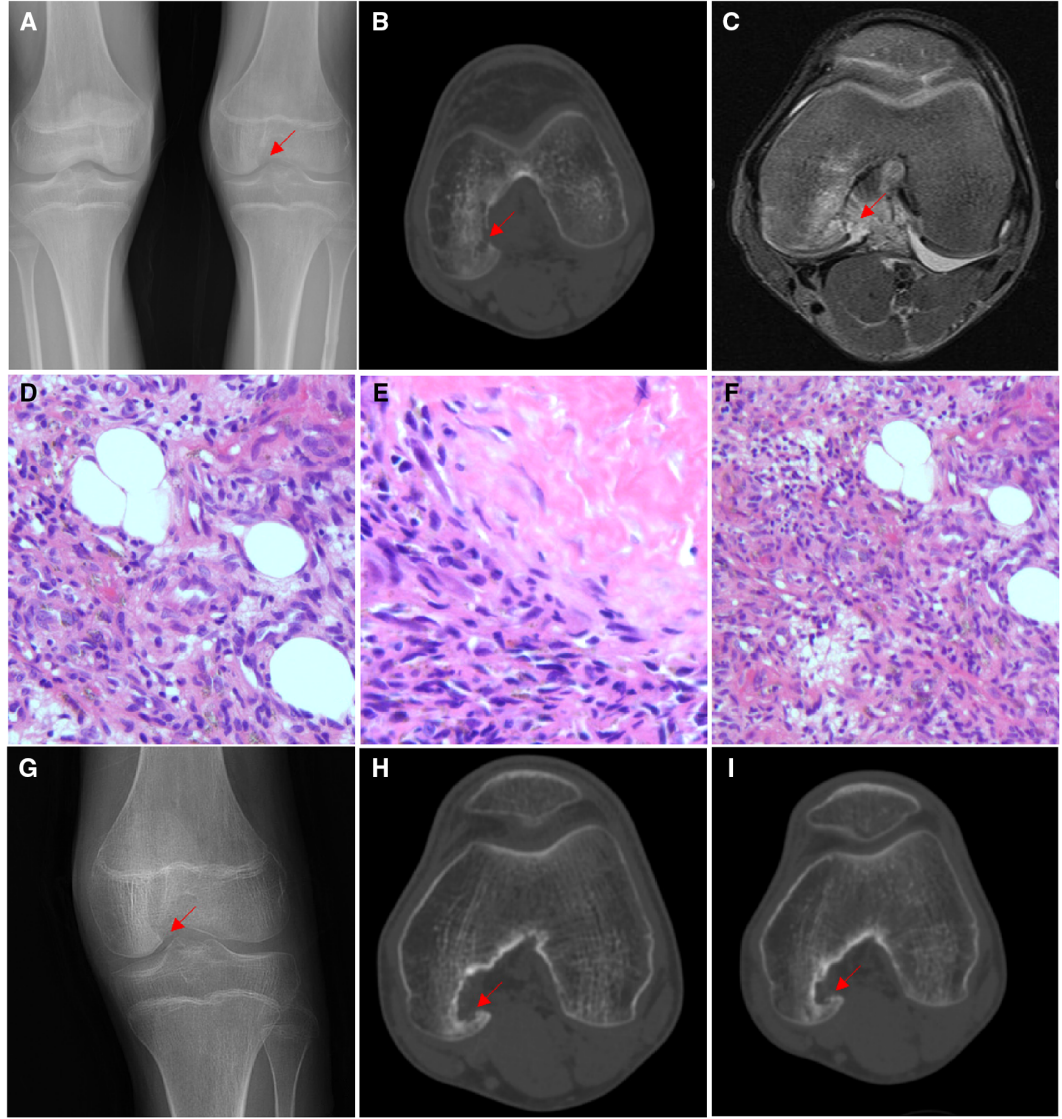
Imaging and pathological features of Case 1. (**A**) X-ray showed bone destruction of the left medial femoral condyle. (**B**) CT showed irregular strips of reduced bone density on the medial side of the left femur that were approximately 1.8 cm*0.5 cm, with increased patchy bone sclerosis around the area and local cortical bone destruction at the inner edge. cMRI showed patchy oedema on the lateral side of the left medial femoral condyle with hyperintensity on T2WI-FS. (**D**) Tumour tissue was distributed in a nodular shape under low-magnification endoscopy, and tumour cells were arranged as onion or concentric circles (HE ×100). (**E**) Mucoid degeneration or hyaline degeneration (HE ×200). (**F**) Tumour tissue intersperses between the trabeculae and occupies the medullary cavity tissue (HE ×100). (**G**) Postoperative x-ray showed bone defect at the posterior edge of the left distal femur. (**H**) CT showed a limited bone defect near the intercondylar fossa of the left femur medial condyle with slight marginal sclerosis 1 year after operation. (**I**) CT results 2 years after the operation were not different from those in panel **H**.

A 15-year-old boy with left knee pain and limited mobility for 10 months was admitted to the hospital. The physical examination revealed that the patient's left knee was slightly swollen, the skin temperature and skin color were normal, the inner space of the knee joint was tender, and the joint movement was restricted. Preoperative x-ray showed bone destruction of the left medial femoral condyle (Figure 1A). CT was performed, and it showed irregular strips of reduced bone density on the medial side of the left femur of approximately 1.8 cm*0.5 cm, with increased patchy bone sclerosis around the area and local cortical bone destruction at the inner edge (Figure 1B). MRI showed patchy oedema on the lateral side of the left medial femoral condyle with hyperintensity on T2WI-FS (Figure 1C). Combined with the clinical manifestations and imaging characteristics, knee joint infection was considered, and arthroscopic removal of the left medial condyle of the femur was performed. However, the pathology after the operation was diagnosed as giant cell angioblastoma (Figures 1D–F), and postoperative review x-ray showed a local bone defect at the distal posterior margin of the left femur (Figure 1H). The patient was followed up for 2 years, and he recovered from the operation well. The CT at 2 years after the surgery showed a limited bone defect near the intercondylar fossa of the left femur medial condyle with slight marginal sclerosis (Figures 1H,I).

### Case 2

**Figure 2:**
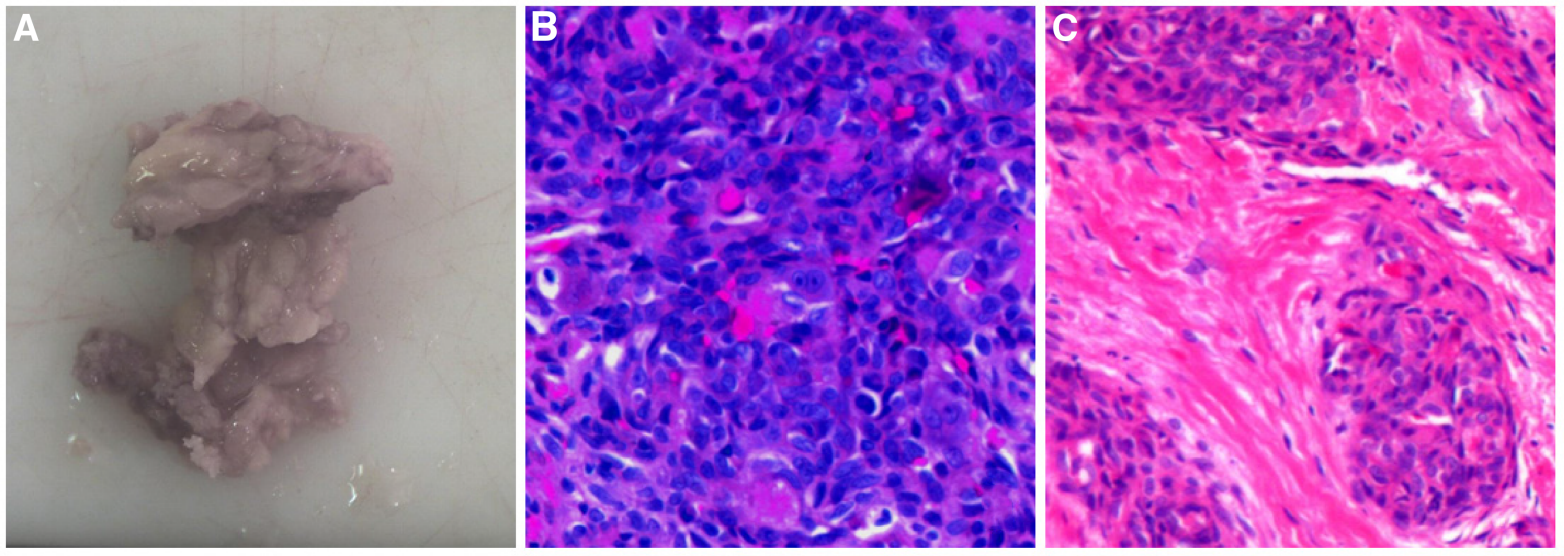
Pathological features of Case 2. (**A**) Macroscopic observation showed greyish-yellow bony tissue of approximately 3 cm*2.5 cm*1 cm. (**B**) Tumour nodules were mainly composed of oval or spindle-shaped vascular endothelial cells under a high-magnification microscope (HE ×400). (**C**) High-magnification microscope showed short spindle-shaped cells in cords and nodules, with local vascular-like lacunae (HE ×200).

A 3-year-old boy with left leg claudication for 2 years was admitted to the hospital. The physical examination showed mild swelling of the lower left thigh and knee, normal skin color, and high temperature with no obvious tenderness. X-ray showed uneven density in the lower segment of the left femur with patchy bone destruction and marked osteosclerosis around the lesion. MRI revealed that the periosteal reaction was detected in the middle and lower parts of the left femur, and the intramedullary cavity density was uneven, which had spread to the epiphyseal area of the distal femur. In the beginning, he was diagnosed with chronic osteomyelitis, but to our surprise, the symptoms did not improve significantly after 2 weeks of continuous antibiotic treatment. Therefore, lesion-removal surgery was performed. Some bones that had been hardened and whitened, and mucus-like tissue were scraped out. Postoperative pathology was diagnosed as giant cell angioblastoma (Figures 2A-C). The patient was followed up for 10 years, and he recovered from the operation well. Unfortunately, his imaging data were absent due to the replacement of a medical record system database.

### Case 3

**Figure 3:**
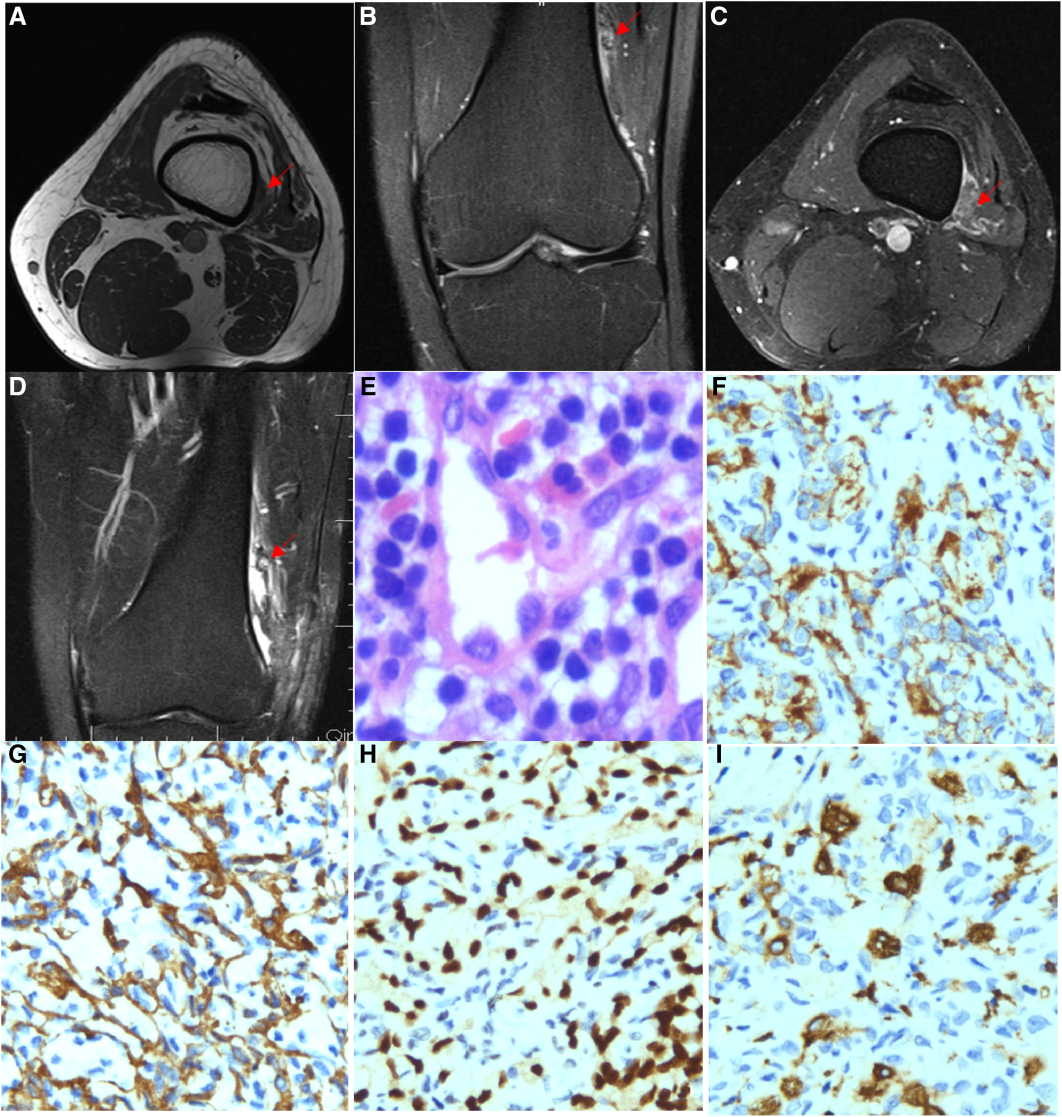
Imaging and pathological features of Case 3. (**A**) MRI showed a patchy abnormal signal shadow in the vastus lateralis while showing an equal or slightly low signal shadow on T1WI. (**B**) T2WI-FS showed a high and low mixed signal shadow and a clear edge, and the enhancement scan was slightly enhanced. (**C**) T2WI-FS series showed a patchy abnormal signal shadow in the vastus lateralis, with a range of approximately 1.5 cm*0.6 cm. No displacement or erosion was observed in the adjacent femur bone, and the bone cortex was intact. (**D**) T2WI-FS series showed a small nodular residual lesion in the vastus lateralis, with soft tissue oedema and a small amount of fluid in the inner margin. (**E**) Lymphocyte aggregation (HE ×400), (**F**) CD31 (IHC ×200) is expressed in endothelial tumour cells. (**G**) SMA (IHC ×200) is expressed in endothelial tumour cells. (**H**) ERG is expressed in endothelial tumour cells (IHC ×200). (**I**) CD163 is expressed in histiocytes (IHC ×200).

A 23-year-old men with recurrent pain on the outside of his left knee joint was admitted to the hospital. The physical examination demonstrated that his left knee was slightly swollen, the lateral part of the joint was obviously tender, and the joint movement was limited. MRI showed a patchy abnormal signal shadow in the vastus lateralis, equal or slightly low signal shadow on T1WI, and high or low mixed signal shadow on T2WI-FS, with clear edges and mild enhancement on an enhanced scan. The proximal femur bone was not advanced or eroded, and the cortical bone was intact (Figures 3A–C). He was considered to be diagnosed with a fibroadenoma or neurogenic tumour and then underwent lesion-removal surgery. However, he was not free from pain after the operation. One month later, an MRI re-examination was performed and it showed that there were small nodular residual lesions in the vastus lateralis, and inner margin soft tissue oedema and a small amount of effusion were also observed (Figure 3D). Lesion resection was performed again, and postoperative pathology was considered giant cell angioblastoma (Figures 3E–I). The patient recovered well in the following 4 years of follow-up.

### Case 4

**Figure 4:**
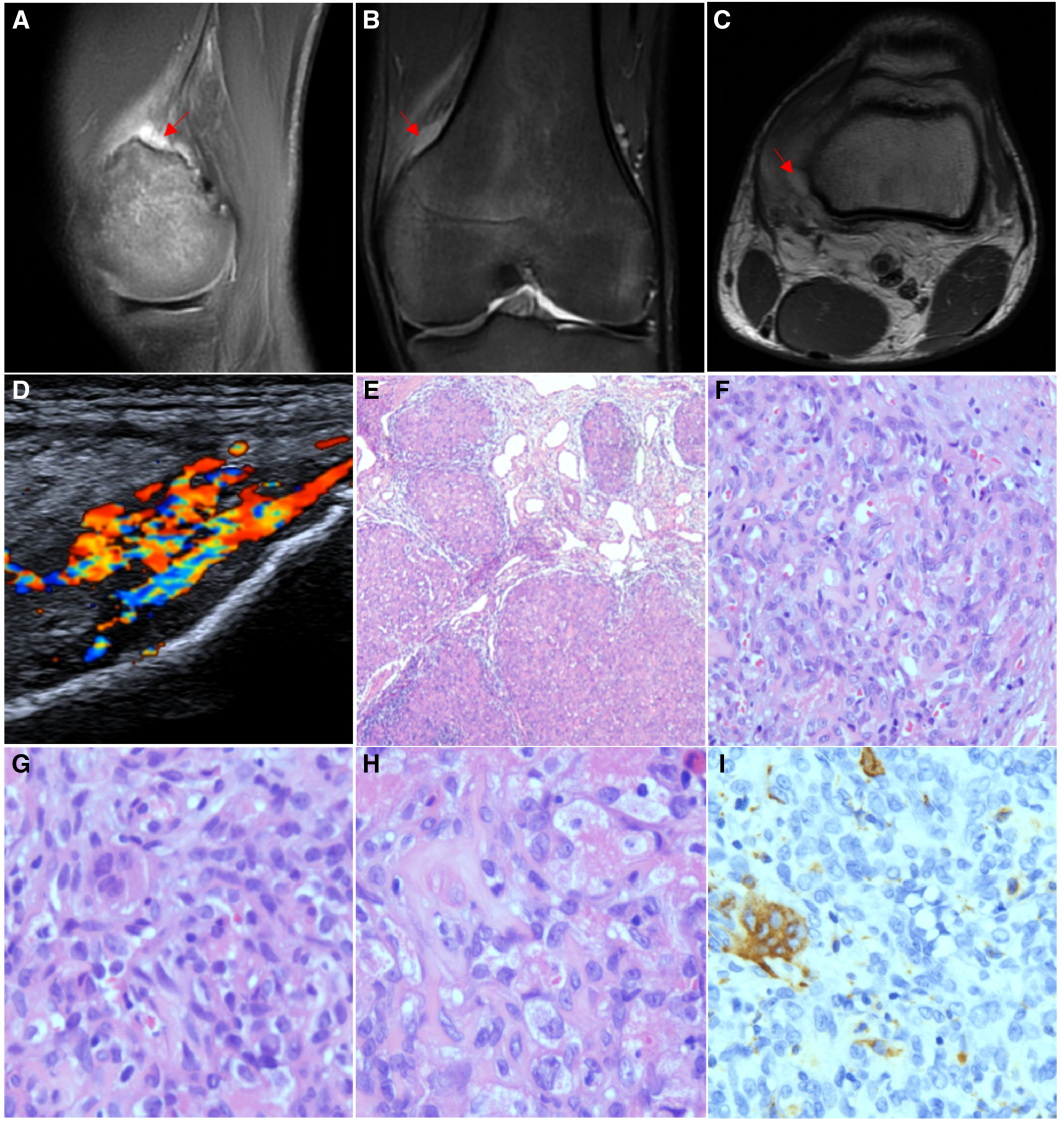
Imaging and pathological features of Case 4. (**A**) MRI showed an abnormal signal shadow between the posterior upper margin of the left medial femoral epicondyle and the vastus medialis. T2WI showed high signal intensity and a slightly unclear boundary, and the enhanced scan showed obvious enhancement. (**B**) Lower part of the vastus medialis was blurred, with patchy T2WI − FS hypersignal oedema, which was indistinctly demarcated from the lesion. (**C**) T1WI + C series shows that the lesion is adjacent to the posterior border of the left medial femoral condyle with local erosion changes by external pressure and a slightly irregular bone cortex in the anterior part. (**D**) Doppler ultrasound showed an irregular 41 mm*11 mm*27 mm hypoechoic mass between the quadriceps femoris muscle and the medial femoral condyle of the left knee with a vague boundary. (**E**) Focal haemangioma signs (HE ×40). (**F**) Microvascular cavities and single-cell cytoplasmic vacuoles were observed between cells (HE ×200). (**G**) Multinucleated osteoclast-like giant cells (HE ×400). (**H**) Foam-like histiocytes (HE ×400). (**I**) CD68 and CD163 are expressed in multinucleated osteoclast-like giant cells (IHC ×200).

An 18-year-old men with left popliteal fossa pain and limited knee movement for 7 years was admitted to the hospital. The physical examination showed obvious muscle atrophy of the left lower limb and swelling of the knee joint with obvious tenderness in the popliteal fossa. The movable joint was painful, and the movement was restricted. MRI showed an abnormal signal shadow between the posterior upper margin of the left medial femoral epicondyle and the vastus medialis. T2WI showed a high signal with a slightly unclear boundary, and an enhanced scan showed obvious enhancement and patchy T2WI-FS high signal oedema shadows with an unclear boundary from the lesion (Figures 4A–C). Doppler ultrasound showed an irregular 41 mm*11 mm*27 mm hypoechoic mass between the quadriceps femoris muscle and the medial femoral condyle of the left knee, with a vague boundary, no capsule, irregular edge, uneven internal echo distribution, and predominantly slow arterial flow (Figure 4D). Considering localized synovitis, a focal resection was performed, and postoperative pathology confirmed giant cell angioblastoma (Figures 4E–I). The patient recovered well at the 3-month follow-up.

## Discussion

GCAB was first reported by Gonzalez-Crussi in 1991, and he proposed the diagnosis of giant cell angioblastoma. To date, 12 cases have been reported, as per our search (1). Due to the limited number of cases, the clinical manifestations, imaging characteristics, pathological features, and prognosis are not clear. Therefore, according to our inference, the true incidence of GCAB may be underestimated. In the newest version of the WHO Classification of Tumours of Soft Tissue and Bone, it is temporarily classified as a middle local aggressive vascular tumour (7). This study reports four new pathologically confirmed cases, increasing the number of reported giant cell angioblastomas to 16. The clinical characteristics of these cases are summarized in Table 1. All patients in this study were male, with an average age of 14.75 years and an average follow-up time of 48.75 months. The longest patient follow-up time was 10 years (Case 2). Two cases involved bone (Cases 1 and 2), and two involved soft tissue (Cases 3 and 4), with lesions located in the lower femur, medial femoral condyle, knee joint, and popliteal fossa. Among these 16 cases, giant cell angioblastoma is more common in infants and children than in adults. The ratio of men to women was 11:5. Eight cases occurred in soft tissues, seven cases occurred in bones, and one case with skin involvement showed erythema, blisters, nodules, infiltrating plaques, or ulcerative changes. The main lesion of GCAB was soft tissue, but seven cases had invaded bone. It is speculated that GCAB may have special biological behaviour and strong invasiveness to bone tissue. The clinical manifestations of cases with soft tissue involvement are mainly increasing masses accompanied by pain or ulcers. On the other hand, cases with bone involvement mainly manifest as discomfort and limited movement of the affected limbs. The course of the disease ranged from birth to 8 years, with the longest case lasting 8 years (Case 3). The patient had never received any drug or other intervention, indicating that GCAB had the biological characteristics of slow growth and an indolent course.

**Table 1:** Clinical summary of patients with giant cell angioblastoma.

Case	Age/sex	Site	Course of disease	Clinical diagnosis	Treatment	Follow-up (months)	Source
1	3 months/F	Right foream	3 months	Fibrohistiocytic tumour	Amputation	Uneventful	González-Crussi et al. (1)
2	7 months/M	Palate	7 months	Giant cell angioblastoma	Subtotal excision, interferon-α	No progression/60	Vargas et al. (2)
3	49 days/M	Right hand	49 days	Vascular tumour	Subtotal excision, interferon-α	No progression/31	Vargas et al. (2)
4	25 days/F	Scalp	25 days	Giant cell angioblastoma	Unavailable	Unavailable	Vargas et al. (2)
5	10 months/M	Palate	3 months	Giant cell angioblastoma	Partial resection, interferon-α 2b	No evidence of disease/72	Marler et al. (3)
6	Neonate/M	Right hand	Innate	Giant cell angioblastoma	Partial resecti-α 2b	No evidence of disease/32	Marler et al. (3)
7	4 years/M	Right knee	1 year	Giant cell angioblastoma	Partial resection	Recurrent/17	Mao et al. (4)
8	41 years/M	Popliteal fossa	4 months	Synovial chondromatosis	Complete resection	No evidence of disease/16	Susanna et al. (5)
9	23 months/F	Right hip joint	7 months	Fibrous dysplasia	Curettage of the lesion	Uneventful/2	Lin et al. (6)
10	8 years/M	Left hip and knee	1 year	Tuberculosis	Focal cleaning	Uneventful/10	Lin et al. (6)
11	37 years/F	Lumbar vertebra	5 months	Tuberculosis	Focal cleaning	Uneventful/21	Lin et al. (6)
12	56 years/F	Left metacarpus, phalange	1 year	Tuberculosis	Focal cleaning	Uneventful/9	Lin et al. (6)
13	15 years/M	Left knee	10 months	Knee infection	Focal cleaning	No evidence of disease/24	Current series, Case 1
14	3 years/M	Left leg	2 years	Giant cell angioblastoma	Curettage of the lesion	No evidence of disease/120	Current series, Case 2
15	23 years/M	Left knee	8 years	Giant cell angioblastoma	Subtotal excision	Recurrent/48	Current series, Case 3
16	18 years/M	Popliteal fossa	7 years	Giant cell angioblastoma	Subtotal excision	Uneventful/3	Current series, Case 4

In terms of imaging features, x-ray and CT scans of those cases with bone involvement showed increased bone density, thickened cortical bone, different degrees of bone destruction, and insignificant swelling of surrounding tissues, which could easily be misdiagnosed as osteomyelitis and tuberculosis. Fortunately, we can distinguish through infection indicators and the mycobacterium tuberculosis test (8, 9). MRI of cases with soft tissue involvement showed irregular, abnormal signal shadows and obvious enhancement, which need to be differentiated from granulomatous diseases, lipomas, or glomus tumours (10–12). Unfortunately, due to the lack of specificity in clinical manifestations or imaging features and adequate understanding of it, clinicians are prone to misdiagnose it as chronic osteomyelitis, bone tuberculosis, synovitis, etc., so they often choose anti-infection or anti-tuberculosis, not surprisingly leading to treatment failure. Therefore, both clinicians and imaging doctors need to have a thorough understanding of GCAB to avoid misdiagnosis and mistreatment. Relying on the obvious morphological characteristics of GCAB, the diagnosis is mainly based on pathological examination and immunohistochemistry. On histological examination, the tumour tissue is nodular, mainly composed of oval or spindle-shaped cells, multinucleated osteoclast-like giant cells, and different-sized blood vessels in interstitials. According to the features of the nodular pattern, multinuclear giant cells and mononuclear histiocytic cells, Mao et al. (4) proposed a way to identify GCAB with giant cell fibroblastoma, angiomatoid fibrous histiocytoma, plexiform fibrous histiocytoma, giant cell angiofibroma, and so on. The immunohistochemistry of the four cases in this study suggested that the vascular endothelial cells in the nodule express CD31, SMA, and ERG. On the other hand, peripheral cells express SMA, while multinucleated osteoclast-like giant cells and histiocytes express CD68 and CD163, which is consistent with the case results reported by Mao et al. (4) and Susanna (5).

At present, there is no optimal treatment for giant cell angioblastoma. The principal treatment is surgery, aiming to remove the tumour as completely as possible and to ensure negative margins. There are no reports or experiences of chemotherapy and radiotherapy after or before the operation. In this study, four patients were treated with surgery, of which three did not relapse during follow-up. One case of a 23-year-old men (Case 3) showed no significant relief of pain after surgery, and an MRI re-examination suggested residual tumour 1 month after the surgery (Figure 1E). He underwent a second operation sooner, experienced relief from the pain, and recovered well during the follow-up for 4 years. Coincidentally, Mao et al. (4) also reported a 4-year-old boy with an invasive disease originating from the bone who also relapsed 17 months after the operation. If the tumour extensively affects the limbs without the necessity of a limb, amputation can be performed. Gonzalez-Crussi et al. (1) reported a case of a 3-month-old girl with giant cell angioblastoma who underwent amputation because of extensive tumour tissue in the forearm soft tissue mass. Although giant cell angioblastoma is an intermediate locally aggressive vascular tumour, most cases have a pattern of slow growth. No distant metastasis or death occurred during the follow-up period in the four cases of this study and the 12 cases reported in the literature. Generally, giant cell angioblastoma is a kind of angiogenic tumour, which provides a theoretical basis for antiangiogenic therapy. Interferons are natural proteins synthesized by white blood cells that have antitumour and antiangiogenic characteristics (13). Vargas et al. (2) and Marler et al. (3) reported that four patients benefitting from antivascular treatment with interferon, suggesting that interferons may be used as adjuvant therapy for giant cell angioblastoma. However, whether our speculation is true requires more cases and studies to support it.

In summary, giant cell angioblastoma is a locally invasive vascular tumour that can develop both in children and adults, although it is more common in males. It can involve skin, mucosal tissues, soft tissues, and bone. The imaging characteristics show bone destruction or irregular, abnormal signal shadows with obvious morphological features. Surgery is now considered the primary treatment, while interferons may be used as an adjuvant treatment for giant cell angioblastoma.
